# Medición de adherencia a antirretrovirales con métodos múltiples en La Romana, República Dominicana

**DOI:** 10.26633/RPSP.2022.207

**Published:** 2022-12-09

**Authors:** Pamela Báez, Adriana Tiburcio, Nicole Alba, Fernando Mateo, Estefani Grullon, Sheyla Cordero, Ana Fernández, Janetly Reinoso, Desireé Cruz, Karina Gómez, Natacha Vargas, Camila Saint-Hilaire, Olga Abreu, Grace Acosta, Mina Halpern, Samantha Stonbraker

**Affiliations:** 1 Clínica de Familia La Romana Pamela Báez La Romana República Dominicana Clínica de Familia La Romana, La Romana, República Dominicana. Pamela Báez; 2 Pontificia Universidad Católica Madre y Maestra Santiago de los Caballeros República Dominicana Pontificia Universidad Católica Madre y Maestra, Santiago de los Caballeros, República Dominicana.; 3 Escuela de Enfermería Universidad de Colorado Colorado Estados Unidos de América Escuela de Enfermería, Universidad de Colorado, Colorado, Estados Unidos de América.

**Keywords:** Adherencia al tratamiento antirretrovirales, VIH, República Dominicana, Adherence, therapeutic, agents, anti-retroviral, HIV, Dominican Republic, Aderência ao tratamento, agente antirretroviral, HIV, República Dominicana

## Abstract

**Objetivo.:**

El objetivo fue desarrollar una herramienta para medir los niveles de adherencia al tratamiento antirretroviral (la TARV) en un entorno de escasos recursos, a partir de la combinación de cuatro métodos de medición de adherencia.

**Métodos.:**

Revisión retrospectiva de 500 expedientes médicos de personas que viven con VIH, elegidos de manera aleatoria desde octubre del 2017 hasta enero del 2020. Se midió la adherencia a la TARV combinando cuatro métodos de medición (porcentaje de cobertura de la TARV recetada, recogida de la TARV en farmacia, nivel de carga viral y autoinforme de adherencia). Se realizaron pruebas de chi al cuadrado con *P* <0,05 para diferencias estadísticamente significativas y regresión binaria logística para identificar probabilidades de adherencia óptima y subóptima. Realizamos pruebas de Spearman para correlación de categorías y alfa de Cronbach para medir la consistencia interna de la herramienta.

**Resultados.:**

Obtuvimos 497 calificaciones de adherencia. De estas, 307 (61,8%) usuarios se calificaron como adherentes, 141(28,4%) como semiadherentes y 49 (9,8%) como no adherentes. Se encontró una mayor probabilidad de adherencia óptima en grupos de 60 años o más (*odds ratio* [OR]: 1,6; IC95%: 0,8-3,5) sin diferencia entre hombres y mujeres (OR: 0,9; IC95%: 0,7-1,4). La prueba de Spearman informó una relación (*r* = 0,8) entre los niveles de carga viral y la calificación final, y la prueba alfa de Cronbach arrojó una modesta consistencia interna (α = 0,7).

**Conclusiones.:**

Se desarrolló una herramienta para medir adherencia en un entorno de escasos recursos. La herramienta presenta niveles modestos de consistencia interna y una correlación fuerte en la categoría de carga viral y adherencia.

## Abstract (en)

**Objective.:**

To develop a tool that measures levels of adherence to antiretroviral treatment (ART) in resource-poor settings, based on a combination of four methods for measuring adherence.

**Methods.:**

Retrospective review of 500 medical records of people living with HIV, randomly selected from October 2017 to January 2020. Adherence to ART was measured by combining four measurement methods (coverage of prescribed ART, ART picked up at pharmacies, viral load, and self-reported adherence). Chi-squared tests were performed with p<0.05 for statistically significant differences and logistic binary regression to identify the probability of optimal and suboptimal adherence. Spearman tests were performed for correlation of categories, and Cronbach’s alpha was used to measure the internal consistency of the tool.

**Results.:**

We obtained 497 adherence scores. Of these, 307 (61.8%) users qualified as adherent, 141 (28.4%) as semi-adherent, and 49 (9.8%) as non-adherent. A higher probability of optimal adherence was found in groups aged 60 years and older (odds ratio [OR]: 1.6; CI95%: 0.8–3.5), with no difference between men and women (OR: 0.9; CI95%: 0.7–1.4). Spearman’s test reported a relationship (r = 0.8) between viral load levels and final score, and Cronbach’s alpha yielded modest internal consistency (α = 0.7).

**Conclusions.:**

A tool was developed to measure adherence to ART in a resource-poor environment. The tool shows modest levels of internal consistency and a strong correlation between viral load and adherence.

## RESUMO

**Objetivo.:**

Desenvolver uma ferramenta para medir os níveis de adesão à terapia antirretroviral (TARV) em um ambiente de poucos recursos, com base na combinação de quatro métodos de medição de adesão.

**Métodos.:**

Revisão retrospectiva de 500 prontuários de pessoas que vivem com HIV, selecionadas aleatoriamente de outubro de 2017 a janeiro de 2020. A adesão à TARV foi medida pela combinação de quatro métodos (porcentagem de cobertura da TARV prescrita, coleta de TARV na farmácia, nível de carga viral e adesão autorrelatada). Foram realizados testes de qui-quadrado com P < 0,05 para diferenças estatisticamente significativas e regressão logística binária para identificar probabilidades de adesão ótima e subótima. Foram realizados testes de Spearman para a correlação de categorias e alfa de Cronbach para medir a consistência interna do instrumento.

**Resultados.:**

Foram obtidos 497 índices de adesão. Entre eles, 307 (61,8%) usuários foram classificados como aderentes, 141 (28,4%) como semiaderentes e 49 (9,8%) como não aderentes. Foi encontrada maior probabilidade de adesão ótima nos grupos de 60 anos ou mais (*odds ratio* [OR]: 1,6; IC95%: 0,8-3,5), sem diferença entre homens e mulheres (OR: 0,9; IC95%: 0,7-1,4). O teste de Spearman constatou uma relação (*r* = 0,8) entre os níveis de carga viral e a pontuação final, e o teste alfa de Cronbach mostrou uma consistência interna modesta (α = 0,7).

**Conclusões.:**

Foi desenvolvida uma ferramenta para medir a adesão em um ambiente de poucos recursos. A ferramenta apresenta níveis modestos de consistência interna e forte correlação de categoria entre carga viral e adesão.

La adherencia médica se define como “a qué extensión el cambio de comportamiento de una persona (…) corresponde con las recomendaciones acordadas con su proveedor de salud” (1). Por ende, mantener una adherencia óptima a la terapia antirretroviral (la TARV) es parte vital del tratamiento para las personas viviendo con el virus de la inmunodeficiencia humana (PVVIH). Sin embargo, no existe un método estándar para medir la adherencia (2), ya sean métodos directos o indirectos (3). Los métodos directos se basan en la medición de marcadores biológicos, ya sea en la sangre o en la orina, o por observación directa de la toma de medicamentos (4). Los métodos indirectos son de índole administrativa (prescripciones y recargas de medicamentos, entre otros) o datos obtenidos de parte del usuario o el proveedor (autoinforme, entrevistas, conteo de pastillas y monitoreo electrónico de medicamentos) (4).

Los autoinformes de adherencia están entre los métodos más utilizados, debido a su bajo costo, una mínima incomodidad para las PVVIH y la administración cómoda (5). Como desventaja, los resultados dependen de la capacidad de PVVIH para recordar su toma de medicamentos y, por deseabilidad del usuario, presentan tendencia al sobreinforme (6, 7). Por otro lado, monitorizar la toma de la TARV es un método que se puede realizar de forma observacional, o través de medios digitales y electrónicos. Las pruebas biométricas en sangre y orina (que miden los niveles de carga viral, el recuento CD y la presencia de metabolitos, entre otros) son métodos cuantificables y demostrables, con alta correlación con los niveles de adherencia (8, 9); las desventajas derivan de su costo y que las informaciones obtenidas se limitan al momento de la toma de muestra (3, 9). Estudios previos señalan que el autoinforme de adherencia puede sobreestimar valores de adherencia en comparación con otros métodos de medición (10). También se ha informado una asociación entre el porcentaje de cobertura de medicamentos y la adherencia cuando se establece un porcentaje de cobertura de la TARV de 95% o más (11).

A nivel mundial, los niveles de adherencia informados pueden variar desde 40% en Laos (12), 50% en India (13), 70% en América Latina y el Caribe (5) y 87% en Nepal (14). En Haití, se notificó que 31% de las personas que recibían la TARV presentaban una adherencia subóptima (15). Por otro lado, en la República Dominicana, una evaluación previa informó que aproximadamente 66% de la población estudiada presentó supresión viral (16), mientras que en Perú, por autoinforme, se calculó una adherencia de 83% de la población que toma la TARV, en su mayoría personas de sexo femenino (8). Asimismo, estudios han asociado mayor adherencia con usuarios de mayor edad y el sexo femenino (5, 15).

La literatura señala que una buena medición de niveles de adherencia solo puede lograrse al combinar diferentes métodos de medición (18, 19). Por ende, se escogieron cuatro métodos para medir la adherencia con el fin de crear una herramienta de medición de adherencia a través de métodos múltiples (MAdAMM). Nuestro objetivo principal fue desarrollar una herramienta para medir los niveles de adherencia a la TARV en un entorno de escasos recursos, a partir de la combinación de cuatro métodos de medición de adherencia. Los objetivos secundarios fueron evaluar correlaciones entre la calificación de adherencia y las categorías de la herramienta y medir la consistencia interna de la escala.

## MATERIALES Y MÉTODOS

Se realizó un estudio de cohorte retrospectivo mediante la aplicación de la MAdAMM, una herramienta diseñada por el equipo de investigación.

### Lugar de estudio

El estudio se realizó en Clínica de Familia La Romana (CFLR), una organización no gubernamental sin fines de lucro que ofrece servicios de atención primaria. En la actualidad, la CFLR tiene la unidad de servicios de atención integral (SAI) para PVVIH más grande en la zona este de la República Dominicana, con aproximadamente 2 500 usuarios activos.

### Herramienta para medir la adherencia

Se utilizó una combinación de cuatro métodos de medición de adherencia para crear la MAdAMM: el porcentaje de cobertura de la TARV recetado, la recogida de la TARV en farmacia, el nivel de carga viral y el autoinforme de adherencia). Cada uno de estos métodos se tradujo en una categoría (A, B, C y D), que se describen a continuación:

**Categoría A para el porcentaje de cobertura de la TARV:** corresponde al porcentaje de cobertura de la TARV recetado entre las visitas médicas, obtenida al comparar la cantidad de la TARV dado en la consulta con la cantidad de días entre las visitas. Esta categoría tiene base en el método de tasa de posesión de medicamentos (20), la cual propone un mínimo de 0,8 de posesión de medicamentos entre una dispensación y otra para calificar como adherente. Se multiplicó la división por 100 para darle presentación de porcentaje. Por ende, se utilizó la siguiente fórmula:

Días de la TARV recetados / días entre una visita y otra ×100 = Porcentaje de cobertura de la TARV

**Categoría B para la recogida de la TARV en farmacia:** refleja la prontitud de recogida de la TARV en el área de farmacia, tomando en cuenta el día de la visita y el día que la TARV fue recogida. Se utiliza la siguiente fórmula:

Fecha en que se recetó la TARV – Fecha en que el usuario recogió la TARV = Prontitud de recogida de la TARV (medidos en días entre la indicación de TARV y la recogida de la TARV)

**Categoría C para el resultado de la carga viral:** representa el resultado más reciente de carga viral (número de copias de virus/mL de sangre) encontrado en los expedientes médicos durante el período de estudio (8, 9). La puntuación por carga viral tiene base en parámetros internacionales: <40 copias/mL equivale a carga viral indetectable; 41-1 000 copias/mL equivale a carga viral suprimida y >1 000 copias/mL equivale a carga viral no suprimida (21).

**Categoría D para el autoinforme de adherencia:** consiste en tres preguntas de autoinforme que aparecen en el expediente médico y a las cuales el paciente responde durante cada consulta médica (“En los últimos 30 días, ¿cuántos días NO tomó al menos una dosis de alguno de sus medicamentos contra el VIH?” presentado en respuesta numérica, “En los últimos 30 días, ¿qué tan bien cumplió con la toma de medicamentos contra el VIH según lo indicado?” con opciones entre ‘Muy mal’ y ‘Excelente’; “En los últimos 30 días, ¿con qué frecuencia ha tomado sus medicamentos con el VIH según lo indicado?” con opciones entre “nunca” y “siempre”) (22, 23). Estas respuestas se almacenaron durante el período de estudio de acuerdo con el cuadro 1, y se calculó un promedio para la calificación final. Se dividió la puntuación en incrementos de 0,5 para evadir la sobrevaloración del autoinforme. Para la categoría D, la puntuación máxima fue 2 puntos, considerando un autoinforme excelente cuando el paciente acumula 1,8 puntos o más (90% de la puntuación); regular con 1,6-1,79 puntos (80% de la puntuación); malo con 1,2-1,59 puntos (60-80% de la puntuación); y terrible con 0-1,1 puntos (<60% de la puntuación).

## Puntuación de la herramienta

Se atribuyó un sistema de puntaje por cada categoría, y estos puntos fueron acumulables para cada usuario hasta obtener una calificación final, cuya puntuación máxima es de 9 puntos (cuadro 1). Para definir la calificación de adherencia, se tomaron en cuenta parámetros que establecen que, con la toma de 90% de sus medicamentos, 80% de los usuarios se presentan con carga viral indetectable (24). Con este parámetro como guía, se calculó que 90% de la puntuación máxima (9 puntos) equivale a 8 puntos, por lo que los usuarios con calificación final de 8 a 9 puntos se calificaron como adherente; los usuarios con calificación final de 6 a 7,99 puntos se calificaron como semiadherente y los usuarios con calificación menor a 6 puntos se calificaron como no adherentes.

**CUADRO 1.** Cálculo de adherencia en usuarios con VIH en la Clínica de Familia La Romana con categorías y puntajes

Categorías	Puntaje
A. Porcentaje de cobertura de la TARV	
≥90% de cobertura	2
80-89% de cobertura	1
<80% de cobertura	0
B. Recogida de la TARV en la farmacia	
TARV recogida el mismo día	2
TARV recogida 1-2 días después	1
TARV recogida >2 días después	0
C. Carga viral	
<40 copias/mL	3^a^
40-1 000 copias/mL	2
>1 000 copias/mL	0
D. Autoinforme	
1. Días sin tomar medicamentos en los últimos 30 días	
0 días	1^b^
1-2 días	0
>2 días	0
2. ¿Qué tan bien tomó los medicamentos en los últimos 30 días?	
Excelente	0,5^b^
Muy bien	0,5
Bien	0
Regular	0
Mal	0
Muy mal	0
No respondió	0
No sabe	0
3. ¿Con qué frecuencia tomó los medicamentos según lo indicado durante los últimos 30 días?	
Siempre	0,5^b^
Casi siempre	0,5
Generalmente	0
A veces	0
Casi nunca	0
Nunca	0
No respondió	0
No sabe	0
Calificación final	
Adherente	8-9 puntos
Semiadherente	6-7,9 puntos
No adherente	≤5,99 puntos

a Puntuación mayor a la categoría C según estudios previos que señalan la correlación entre métodos directos y medida de adherencia (8, 9, 25).

b Se dividió la puntuación de la categoría D con base en notificaciones de sobrevaloración de adherencia a través del autoinforme (10, 22, 26).

### Población y recopilación de datos

Para medir la adherencia a la TARV con MAdAMM, se realizó una selección aleatoria de 100 PVVIH en cinco ocasiones diferentes, con recopilación de la información sobre la adherencia de cada usuario durante un período de seis meses. Las cinco muestras correspondieron al período comprendido entre octubre del 2017 y enero del 2020. Para cada recopilación, se incluyeron expedientes pertenecientes a 100 usuarios activos en el programa del SAI, con edad mayor a 18 años y quienes habían iniciado la TARV por un período mayor a seis meses antes al momento de identificar la muestra.

En cada recopilación, la muestra se eligió de forma aleatoria simple usando la siguiente fórmula:

X/100 = Número aleatorio para selección de expediente

Donde X es el número de usuarios activos en el período de recolección. Por lo tanto, si se encuentran 2 400 expedientes de PVVIH activos en el SAI al iniciar una recopilación de datos para una muestra, se calcula 2 400/100 = 24.

Por ende, se seleccionó un usuario cada 24 expedientes dentro de la población para la recopilación de datos, hasta llegar a 100 expedientes. Estos expedientes se organizan de manera numérica, de menor a mayor, y en ese sentido se localizaron para la recopilación. Durante la revisión de expedientes, se completaron los datos indicados por MAdAMM en una planilla de Excel^®^ (cuadro 1). En total, se extrajeron informaciones de 500 expedientes dentro de los períodos estudiados y, luego de omitir usuarios con datos faltantes, se obtuvieron 497 calificaciones finales. Para ser omitidos, se tomó como criterio la falta de puntuación final en alguna de las categorías para medir la adherencia.

### Análisis de datos

Se utilizó el análisis descriptivo para el sexo y la edad de la población, cada una de las categorías y para la distribución de adherencia entre los PPVIH con calificaciones finales. Luego se realizaron tabulaciones de chi al cuadrado para analizar las diferencias entre el sexo y la edad de la población, las cuatro categorías de la herramienta y por igual con la distribución de adherencia. Se establecieron diferencias estadísticamente significativas con *P* <0,05.

Por último, se recodificó la calificación final en una variable dicótoma llamada adherencia óptima, donde la adherencia óptima es representada por la calificación “adherente” = 8 puntos o más y la adherencia subóptima es representada por las calificaciones “semiadherente” y “no adherente” = menos de 8 puntos. Esta variable dicótoma se usó para la regresión logística binaria con relación al sexo y la edad de las PVVIH y las cuatro categorías de MAdAMM, y se estableció la probabilidad de adherencia óptima.

Además, se realizó el análisis de la correlación entre la calificación final y las distintas categorías (A, B, C y D), por lo que usamos la prueba de rangos de Spearman (*r*), y se realizó la prueba de alfa de Cronbach (α) para medir la consistencia interna. Para le análisis de los datos se utilizó el *software* Stata^®^ versión 16 para los análisis.

### Consideraciones éticas

El protocolo fue aprobado por el comité de ética de la Pontificia Universidad Católica Madre y Maestra y el Consejo Nacional de Bioética de la República Dominicana. Se cumplió con todos los requisitos de la organización y de los dos comités de ética para mantener la confidencialidad de los datos. Debido a que estos se recopilaron a partir de expedientes médicos, no fue necesario el consentimiento informado de los participantes. Para mantener la privacidad de los pacientes, se utilizó un código numérico para cada participante y se mantuvieron los datos en una base de datos en un archivo encriptado con contraseña.

## RESULTADOS

### Demográficos

Se incluyó un total de 497 usuarios en el análisis. Según el sexo, 278 (56,1%) expedientes fueron de sexo femenino y 218 (43,9%) de sexo masculino (cuadro 2). La edad media fue de 44 años (deviación estándar [DE] ± 11,9). Según la edad, 33,9% de la muestra tenía entre 40 y 49 años (n = 168), seguido por los grupos de 30 a 39 años (113; 22,8%), 50 a 59 años (102; 20,5%), 60 años o más (61; 12,3%) y 18 a 29 años (52; 10,5%).

### Puntuación de las PVVIH según la MAdAMM

**Categoría A:** de 497 usuarios, 413 (83,3%) presentaron un porcentaje de cobertura de la TARV de 95% o más, 55 (11,1%) usuarios presentaron un porcentaje de cobertura de 80-94%, y 28 (5,6%) presentaron porcentaje de cobertura de la TARV menor de 80% (cuadro 2). No se encontraron diferencias estadísticamente significativas entre la cobertura de la TARV por sexo ni por edad (*P* >0,05).

**Categoría B:** en los períodos estudiados, 398 (79,5%) recogidas de la TARV de la farmacia se realizaron el mismo día de la visita médica, 81(16,6%) prescripciones se recogieron un día después, y 18 (3,9%) se recogieron 2 días o más después de la consulta médica. No se encontraron diferencias estadísticamente significativas entre la prontitud de recogida de la TARV por sexo ni por edad (*P* >0,5). En el cuadro 3 se muestra el análisis bivariable.

**Categoría C:** más de la mitad de los usuarios presentaron una carga viral indetectable (n = 262; 53,8%), 183 presentaron una carga viral de entre <40 a 1 000 copias/mL (37,6%) y 42 (8,6%) resultados mostraron una carga viral >1 000 copias/mL. Al realizar análisis bivariable (cuadro 3) no se encontraron diferencias estadísticamente significativas por sexo ni por edad (*P* >0,05).

**CUADRO 2.** Distribución demográfica de los participantes y de las categorías de la medición de adherencia a través de métodos múltiples

Variable	N (%)
Sexo	
Masculino	218 (43,9)
Femenino	278 (56,1)
Grupos de edad (años)	
18-29	52 (10,5)
30-39	113 (22,8)
40-49	168 (33,9)
50-59	101 (20,5)
60 o más	61 (12,3)
Categoría A. Porcentaje de cobertura de la TARV	
95% o más	413 (83,3)
80-94%	55 (11,1)
79% o menos	28 (5,6)
Categoría B. Recogida de la TARV en la farmacia	
El mismo día	398 (79,5)
1 día después	81 (16,6)
2 días o más después	18 (3,9)
Categoría C. Carga viral (copias/mL)	
No detectable	262 (53,8)
<40-1 000 copias	183 (37,6)
>1 000 copias	42 (8,6)
Categoría D. Autoinforme de adherencia	
Terrible	18 (3,6)
Malo	29 (5,8)
Regular	40 (8,1)
Excelente	410 (82,5)

***Fuente:*** elaborado a partir de los resultados del análisis descriptivo.

**Categoría D:** de los participantes seleccionados, 410 (82,5%) autoinformaron una excelente adherencia, 40 (8,1%) informaron una adherencia regular, 29 (5,8%) como mala y 18 (3,6%) como terrible. No se encontraron diferencias estadísticamente significativas de autoinforme por sexo ni por edad (*P* >0,05).

**CUADRO 3.** Distribución de calificación de adherencia por sexo, edad y categorías de MAdAMM (n = 497)

	Adherente N (%)	Semiadherente N (%)	No adherente N (%)	Totales	Valor de *P*
Distribución de adherencia	307 (61,8)	141 (28,4)	49 (9,8)	497	-
Sexo					
Masculino	133 (61,0)	65 (29,8)	20 (9,2)	218	0,783
Femenino	173 (62,2)	76 (27,3)	29 (10,4)	278	
Edad (años)					
18-29	29 (55,8)	17 (32,7)	6 (11,5)	52	0,589
30-39	71 (62,8)	27 (23,9)	15 (13,3)	113 (22,8)	
40-49	104 (61,9)	47 (28,0)	17 (10,1)	168 (33,9)	
50-59	60 (59,4)	32 (31,7)	9 (8,9)	101 (20,4)	
60 o más	41 (67,2)	18 (29,5)	2 (3,3)	61 (12,3)	
Categoría A. Porcentaje de cobertura de la TARV					
≥95%	281 (68,0)	104 (25,2)	28 (6,8)	413 (83,3)	<0,0001
80-94%	20 (36,4)	24 (43,6)	11 (20)	55 (11,1)	
<80%	5 (17,9)	13 (46,4)	10 (35,7)	28 (5,6)	
Categoría B. Recogida de la TARV en la farmacia					<0,0001
El mismo día	307 (100)	129 (91,5)	38 (77,6)	474 (95,4)	
Un día después	0 (0)	12 (8,5)	11 (22,5)	23 (4,6)	
Categoría C. Resultado de la carga viral					
No detectable	236 (88,4)	30 (11,2)	1 (2,0)	267 (53,3)	<0,001
<40-1 000 copias	71 (38,8)	104 (56,8)	8 (4,4)	183 (36,8)	
1 000 copias o más	0 (0)	7 (14,9)	40 (85,1)	47 (9,5)	
Categoría D. Autoinforme					
Terrible	2 (11,1)	10 (55,6)	6 (33,3)	18 (3,6)	<0,001
Malo	4 (13,8)	14 (48,3)	11 (37,9)	29 (5,8)	
Regular	10 (25)	25 (62,5)	5 (12,5)	40 (8,0)	
Excelente	307 (61,8)	92 (22,4)	27 (6,6)	410 (82,5)	

*Fuente:* elaborado a partir de los resultados obtenidos con la prueba de chi cuadrado.

### Calificación de adherencia

De 497 usuarios, 307 (61,8%) se calificaron como adherentes, 141 (28,4%) calificaron como semiadherentes y 49 (9,8%) como no adherentes. Según el sexo, 62,2% de las mujeres y 61% de los hombres calificaron como adherentes (cuadro 2). Para ambos sexos, aproximadamente 10% calificaron como no adherentes (9,2% fueron del sexo masculino y 10,4%, del sexo femenino). No se encontraron diferencias estadísticamente significativas en cuanto al sexo y el nivel de adherencia (*P* >0,05).

Por grupo de edad, el mayor porcentaje de usuarios con calificación adherente fue el grupo de edad de 60 años o más (n = 41; 67,2%), seguido por el grupo de 30-39 años (n = 71; 62,8%). La menor distribución de la población adherente (n = 29; 55,8%) se observó en el grupo de 18-29 años (cuadro 3). En cuanto a los usuarios no adherentes, la mayor distribución por edad fue entre el grupo de 30-39 años (n = 15; 13,3%). No se encontraron diferencias estadísticamente significativas entre la adherencia por grupos de edad (*P* >0,05).

### Regresión binaria

No se encontraron diferencias estadísticamente significativas en las probabilidades de adherencia óptima entre hombres y mujeres (OR: 0,95; IC95%: 0,6-1,4 para el sexo masculino), mientras que todas las edades tuvieron mayor probabilidad de informar una adherencia óptima que el rango de edad de 18-29 años, con la mayor probabilidad el grupo de 60 años o más (OR: 1,6; IC95%: 0,8-3,5). Sin embargo, no se encontraron diferencias estadísticamente significativas en estos análisis de probabilidad por edad (*P* = 0,2) ni sexo (*P* = 0,8). Por otro lado, se encontró que el grupo con porcentaje de cobertura de la TARV menor a 80% presenta menor probabilidad de adherencia óptima (OR: 0,1; IC95%: 0,04-0,3) en comparación con una cobertura de 95% o más, y menor probabilidad de calificación de adherencia óptima para usuarios con carga viral detectable (OR: 0,1; IC95%: 0,1-0,1) que en PVVIH con resultado no detectable en su carga viral (cuadro 4). Para estas categorías, las diferencias fueron estadísticamente significativas (*P* <0,01 para ambas categorías).

**CUADRO 4.** Regresión logística binaria para calificar al usuario como adherente, por sexo, edad y por categorías (A, B, C y D) (n = 495)

	Razón de probabilidad	Error estándar	Valor de *P*	IC95%	
Sexo					
Femenino (referencia)					
Masculino	0,95	0,18	0,78	0,66	1,37
Edad (años)					
18-29 años (referencia)					
30-39	1,34	0,46	0,39	0,69	2,61
40-49	1,29	0,4	0,43	0,69	2,42
50-59	1,16	0,40	0,67	0,59	2,28
60 o más	1,63	0,63	0,21	0,76	3,49
Categoría A. Porcentaje de cobertura de la TARV					
≥95% (referencia)					
80-94%	0,27	0,08	0,00	0,15	0,48
≤83%	0,10	0,05	0,00	0,04	0,27
Categoría B. Recogida de la TARV^a^					
Categoría C. Carga viral					
No detectable (referencia)					
40-1 000 copias/mL	0,08	0,20	0,000	0,05	0,13
≥1 000 copias/mL o más^a^					
Categoría D. Autoinforme					
Terrible (referencia)					
Malo	0,25	0,92	0,79	-1,56	2,01
Regular	0,98	0,76	0,24	-0,65	2,62
Excelente	2,97	0,76	0,00	1,49	4,46

*Fuente*: elaborado a partir de los resultados obtenidos por regresión logística binaria.

a Resultados de regresión logística binaria no disponible por colinearidad de los datos. IC95%, intervalo de confianza del 95%; TARV, terapia antirretroviral.

### Resultados de confiabilidad, validación y correlación entre métodos de medición y calificación final

Al utilizar la prueba alfa de Cronbach (cuadro 5), se obtuvo una consistencia interna de α = 0,7 entre las categorías, con la mayor correlación ítem-prueba presentada por el resultado de carga viral en relación con el nivel de adherencia (α = 0,8). Se analizó la correlación de los métodos de medición con el resultado de la calificación final a través de la correlación de Spearman. Para la calificación final de adherencia, la correlación más fuerte fue con el resultado de la carga viral (*r* = −0,8), la cual fue la única prueba con fiabilidad entre las categorías. Los resultados de las demás categorías fueron el porcentaje de cobertura de la TARV (*r* = 0,5), el autoinforme (*r* = 0,4) y la prontitud en la búsqueda de los la TARV en farmacia (*r* = 0,4).

**CUADRO 5.** Resultados de correlación con la prueba de Spearman y de fiabilidad con la prueba de alfa de Cronbach

Pruebas	Categoría A: porcentaje de cobertura de la TARV	Categoría B: recogida de la TARV en la farmacia	Categoría C: resultado de la carga viral	Categoría D: autoinforme
Prueba de Spearman	0,49	0,43	-0,79	0,454
Prueba de alfa de Cronbach				
Correlación ítem-prueba	0,57	0,36	0,77	0,55
Correlación entre categorías	0,30	0,25	0,51	0,55
Prueba alfa de Cronbach	0,66	0,68	0,55	0,65
Promedio de covariancia	0,08			
Promedio de la prueba alfa de Cronbach	0,66

*Fuente:* elaborado a partir de los resultados obtenidos con la prueba de Spearman y la prueba alfa de Cronbach.

TARV, terapia antirretroviral.

## DISCUSIÓN

La meta principal de este estudio fue crear una herramienta (MAdAMM) para medir la adherencia a terapia antirretroviral en CFLR, una institución con escasos recursos. Se encontró que 61,8% de la población calificaba como adherente. Sin embargo, no se encontraron asociaciones significativas entre la edad o el sexo y la calificación de adherencia. Mediante pruebas de rango de Spearman y alfa de Cronbach, se encontró una consistencia interna modesta (α = 0,7) entre las categorías y una buena correlación (*r* = −0,8) entre la carga viral y la calificación de adherencia, la única prueba con fiabilidad entre las categorías.

Con respecto a la edad, los menores niveles de adherencia se observaron en usuarios de menor edad, así como una mayor adherencia en el grupo etario de 60 años o más. Estos resultados de nivel de adherencia concuerdan con notificaciones previos en República Dominicana (16). De la misma manera, la mayor distribución de adherencia en usuarios de mayor edad coincide con la encontrada en estudios de Mozambique (27), Haití (15), Brasil (28) y a través de revisión literaria (5, 29). Sin embargo, no se encontraron diferencias estadísticamente significativas entre hombres y mujeres en nuestro estudio. En cuanto a las propiedades de la MAdAMM, se encontró una correlación fuerte y de dirección negativa (*r* = −0,8) entre los resultados de carga viral y la calificación de adherencia, junto con correlaciones menos fuertes y de dirección positiva entre la adherencia y las demás categorías. Este resultado se explica porque la medición de carga viral es el único método directo para medir la adherencia dentro de la MAdAMM (8, 9). Cabe notar que la prueba de correlación entre la calificación de adherencia y el autoinforme (*r* = 0,5) fue comparable con la relación entre la calificación de adherencia y la prontitud de recogida de la TARV en la farmacia (*r* = 0,4), pero menor que el porcentaje de la cobertura de la TARV entre las visitas (*r* = 0,5); este resultado también se reflejó en la prueba de Cronbach. Esto se podría explicar por el efecto de la parcialidad de memoria y deseabilidad por parte de los usuarios, que presentan tendencia a sobrenotificar la adherencia al personal médico (5, 30).

Tanto en la prueba de Spearman como en la de alfa de Cronbach, la categoría con mayor fiabilidad y relación categoría-resultado fue el resultado de carga viral, que también coincide con los resultados previos encontrados en la literatura (8). El resultado subóptimo (α = ≤0,7) de la prueba de fiabilidad de Cronbach significa que hay que interpretar resultados con precaución. Sin embargo, la coincidencia de los resultados aquí presentados con resultados previos de niveles de adherencia informado (5) es un buen indicador de la funcionabilidad de MAdAMM. Para futuros proyectos, la consistencia interna podría potencializarse al añadir variables que sean métodos de medición de adherencia, como valores de CD4, conteo de pastillas o porcentaje de citas cumplidas.

Una limitación del estudio consiste en que solo se tomó la muestra enfocada en la adherencia a la TARV en PVVIH, por lo que los resultados pueden no ser generalizables a otras enfermedades. De la misma manera, no se indagaron facilitadores y barreras asociados a la adherencia farmacológica de los usuarios, por lo que se dificulta entender los factores asociados a la aparición de adherencia subóptima dentro de la población estudiada.

A pesar de las limitaciones, la MAdAMM puede ser valiosa para medir adherencia a la TARV, especialmente en entornos de escasos recursos sin acceso a *software* de análisis de datos estadísticos especializados o conocimiento de cómo hacer las estadísticas apropiadas. La recomendación se basa en las fortalezas encontradas en el estudio, ya que la MAdAMM es innovadora en el sentido de que combina métodos objetivos y con base en las informaciones de los usuarios y toma en cuenta las ventajas y desventajas a la hora de medir la adherencia. La MAdAMM es un instrumento creado en Microsoft Excel^®^, por lo que es accesible a varios tipos de entornos, fácil de usar, reproducible y con bajo costo de aplicación. Al ser un método retrospectivo que usa expedientes de los usuarios, no interrumpe el flujo y la dinámica de la cita médica de los usuarios, ni implica riesgos de incomodar al usuario durante su visita, y permite el análisis de diferentes períodos de tiempo y tamaños mayores de poblaciones. Además, se resguarda la confidencialidad y privacidad del usuario al no usar ninguna información identificable de los pacientes. Sin embargo, al utilizar la MAdAMM se recomienda la capacitación rigurosa del equipo médico que realiza el autoinforme, el personal de farmacia que dispensa los medicamentos y el equipo encargado de la recopilación y la entrada de datos, ya que de estos datos depende la calificación final. Otro potencial uso deriva de la adaptabilidad de la MAdAMM; esta herramienta se puede ajustar para incluir otras variables que actúen actuar como predictores al realizar mediciones de adherencia en su entorno clínico.

## Conclusiones

La MAdAMM se desarrolló para medir la adherencia a la TARV en un entorno de escasos recursos. Los resultados muestran niveles de adherencia que concuerdan con la literatura encontrada y, al combinar diferentes métodos cuantificables tantos directos como indirectos, existe fiabilidad sobre la distribución de adherencia dentro de la población estudiada. La MAdAMM presenta niveles modestos de consistencia interna y una correlación fuerte en la categoría de carga viral y adherencia.

Los resultados indican que la MAdAMM podría ser útil en otros entornos de escasos recursos para medir la adherencia de otras PPVIH. Para probar su validez externa, se sugiere que otras organizaciones y grupos de investigación usen y prueben esta herramienta. También puede ser beneficioso combinar la MAdAMM con la investigación cualitativa, lo cual podría mejorar considerablemente la comprensión del comportamiento de las PVVIH con la meta de identificar barreras y facilitadores de la adherencia a la TARV, con informaciones basadas en la evidencia tomada de la población aquí estudiada.

## Declaración.

Las opiniones expresadas en este manuscrito son responsabilidad del autor y no reflejan necesariamente los criterios ni la política de la *RPSP/PAJPH* y/o de la OPS.
